# Dual data and motif clustering improves the modeling and interpretation of phosphoproteomic data

**DOI:** 10.1016/j.crmeth.2022.100167

**Published:** 2022-02-14

**Authors:** Marc Creixell, Aaron S. Meyer

**Affiliations:** 1 Department of Bioengineering, University of California, Los Angeles, Los Angeles, CA 90024, USA; 2 Department of Bioinformatics, University of California, Los Angeles, Los Angeles, CA 90024, USA; 3 Jonsson Comprehensive Cancer Center, University of California, Los Angeles, Los Angeles, CA 90024, USA; 4 Eli and Edythe Broad Center of Regenerative Medicine and Stem Cell Research, University of California, Los Angeles, Los Angeles, CA 90024, USA; 5 Lead contact

## Abstract

Cell signaling is orchestrated in part through a network of protein kinases and phosphatases. Dysregulation of kinase signaling is widespread in diseases such as cancer and is readily targetable through inhibitors. Mass spectrometry-based analysis can provide a global view of kinase regulation, but mining these data is complicated by its stochastic coverage of the proteome, measurement of substrates rather than kinases, and the scale of the data. Here, we implement a dual data and motif clustering (DDMC) strategy that simultaneously clusters peptides into similarly regulated groups based on their variation and their sequence profile. We show that this can help to identify putative upstream kinases and supply more robust clustering. We apply this clustering to clinical proteomic profiling of lung cancer and identify conserved proteomic signatures of tumorigenicity, genetic mutations, and immune infiltration. We propose that DDMC provides a general and flexible clustering strategy for the analysis of phosphoproteomic data.

## In brief

Measuring cell signaling by mass spectrometry-based phosphoproteomics provides a promising opportunity to direct cancer therapy development. Here, we present dual data motif clustering (DDMC), a clustering and kinase prediction strategy that identifies signaling nodes by grouping phosphosites according to their phosphorylation signal and amino acid sequence.

## Graphical Abstract

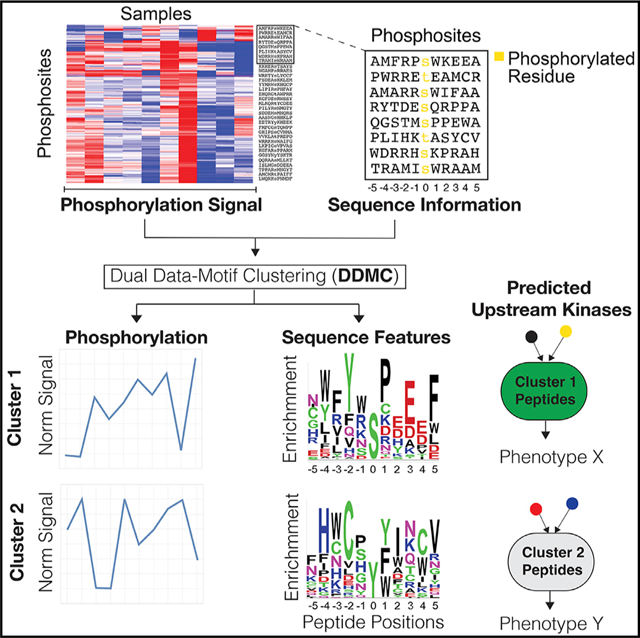

## INTRODUCTION

Cell signaling networks formed by protein kinases dictate cell fate and behavior through protein phosphorylation, including in diseases such as cancer (Hunter, 1995). Measuring cell signaling by mass spectrometry (MS)-based global phosphoproteomics provides a promising opportunity to direct therapy development (Yaffe, 2019), particularly given the accessibility of these signaling changes to drug targeting. Nevertheless, despite the rapid accumulation of large-scale phosphoproteomic clinical data, it is still difficult to link signaling events leading to observed proteomic alterations and phenotypic outcomes.

One approach to analyze phosphoproteomic measurements has been to infer the activity of upstream kinases. For instance, kinase-substrate enrichment analysis averages the signals of groups of known kinase substrates to infer enriched pathways in biological samples (Casado et al., 2013). Another method, integrative inferred kinase activity (INKA), infers kinase activity by integrating the overall and activation loop phosphorylation of kinases alongside the phosphorylation abundance of known substrates. Kinase-substrate relationships are either experimentally determined or predicted by NetworKIN, an algorithm that uses sequence motif and protein-protein network information (Linding et al., 2007; Beekhof et al., 2019; Hornbeck et al., 2019). Finally, Scansite predicts kinase-substrate interactions using sequence motifs generated from oriented peptide library scanning experiments (Obenauer et al., 2003). These methods, sometimes in combination, help to reconstruct signaling pathway activities from individual samples.

However, due to several limitations, kinase-substrate inference still provides a limited view of signaling network changes. Kinase prediction methods are necessarily dependent on having well-characterized kinase-substrate interactions, but most of the phosphoproteome remains largely uncharacterized (Needham et al., 2019). Just 20% of kinases have been shown to phosphorylate 87% of currently annotated substrates, and around 80% of kinases have fewer than 20 substrates, with 30% yet to be assigned a single substrate (Needham et al., 2019). Insights dependent on this unequal knowledge distribution are less likely to identify understudied protein kinases. An additional major challenge, particularly with discovery-mode multiplexed tandem mass tag (TMT) MS, is missing values. The technique processes batches of samples with stochastic coverage in each experiment. This means that the portion of the phosphoproteome quantified in the samples of different TMT experiments varies (Tabb et al., 2010). Computational tools usually require complete datasets, and so data are frequently preprocessed by imputing missing values—inflating the effect of certain measurements or throwing out any peptides displaying missing values—at the expense of losing critical information (Chen et al., 2020; Gillette et al., 2020). Kinase enrichment and prediction methods are further compromised by this problem.

Clustering methods, such as hierarchical clustering or k-means, can be used to cluster phosphopeptides based on similarities in the patterns of their abundance (Mertins et al., 2016; Chen et al., 2020; Deb et al., 2020). This clustering criterion results in groups of peptides that display similar phosphorylation patterns across conditions, but that may be targeted by sets of different upstream kinases that are not directly inferred by these methods. The residues surrounding phosphorylation sites have evolved to become fine-tuned motifs that confer signaling specificity and fidelity (Zarrinpar et al., 2003; Tan et al., 2009). Clustering based on motif similarity might, therefore, improve model interpretation by facilitating the identification of upstream kinases modulating clusters that display conserved sequence motifs. On the other hand, clustering peptides based on sequence alone may result in groups of proteins that, while sharing the same set of upstream kinases, are differently regulated due to context. We therefore hypothesized that combining phosphorylation status and sequence similarity may enable a balanced characterization of the cell signaling state.

**Figure 1.**
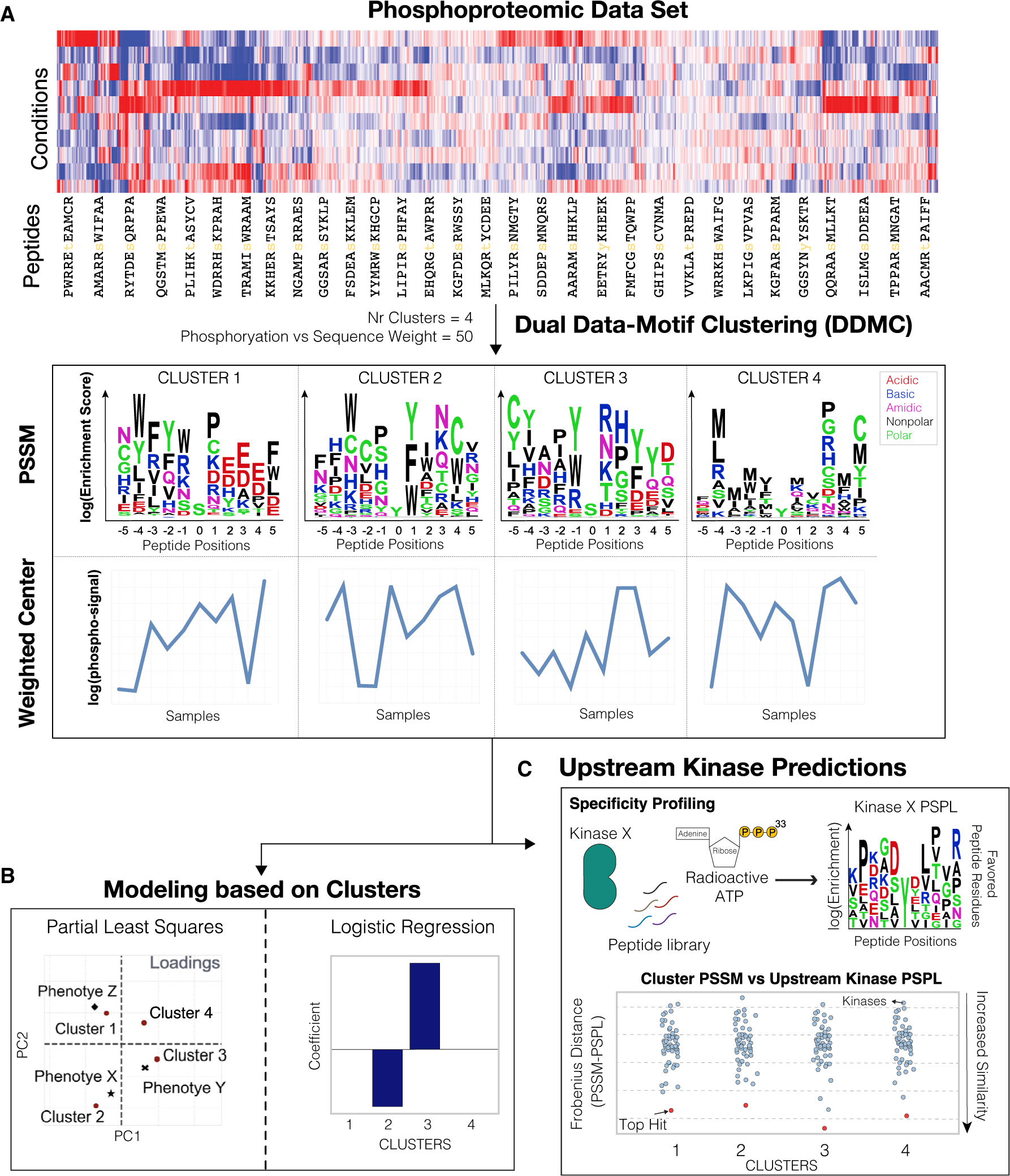
Schematic of the DDMC approach to cluster global signaling data and infer upstream kinases driving phenotypes (A) DDMC is run to cluster an input phosphoproteomic dataset to generate four clusters of peptides that show similar sequence motifs and phosphorylation behavior. (B) Predictive modeling using clusters allows one to establish associations between specific clusters and features of interest. (C) Putative upstream kinases regulating clusters can be predicted by comparing the experimentally generated specificity profiles of upstream kinases (kinase PSPL) and the cluster PSSMs PSSM; Position-specific scoring matrix, PSPL; Position scanning peptide library (Hutti et al., 2004; Begley et al., 2015). See also Figures S1 and S2.

Here, we present an algorithm known as dual data and motif clustering (DDMC) that probabilistically and simultaneously models both the peptide phosphorylation variation and peptide sequence motifs of peptide clusters to reconstitute cell signaling networks (Figure 1). A key distinction of DDMC is that it analyzes multidimensional data, whereas kinase enrichment tools operate on individual samples, relying on prior knowledge. Importantly, DDMC clusters are not limited to pre-existent kinase motifs and therefore do not rely on previous kinase-substrate characterization. Thus, DDMC kinase predictions can lead to the association of understudied kinases and phenotypic responses. We propose that DDMC represents a unified alternative that overcomes fundamental methodologic issues of current tools. To test the utility of our method, we analyzed the phosphoproteomes of 110 treatment-naïve lung adenocarcinoma (LUAD) tumors and 101 paired normal adjacent tissues (NATs) from the National Cancer Institute (NCI)’s Clinical Proteomic Tumor Analysis Consortium (CPTAC) LUAD study (Gillette et al., 2020). We characterized the phosphoproteome of patients by identifying those signaling signatures associated with tumorigenesis, the presence of specific mutations, and tumor immune infiltration. In total, we demonstrated DDMC as a general strategy for improving the analysis of phosphoproteomic surveys.

## RESULTS

### Constructing an expectation-maximization algorithm tailored for clustering phosphoproteomic data

In seeking to cluster phosphoproteomic measurements, we recognized that these data provide two pieces of information: the exact site of phosphorylation on a peptide sequence and some measure of abundance within the measured samples. Both pieces of information are critical to the overall interpretation of the data. Based on this observation, we built a mixture model that probabilistically clusters phosphosites based on both their peptide sequence and abundance across samples (Figure S1). In each iteration, DDMC applies an expectation-maximization algorithm to optimize clusters that capture the average features of member sequences and their abundance variation (Figures 1A and S1). Both information sources—the peptide abundance and sequence—can be prioritized during cluster fitting by a weight parameter. With a weight of 0, DDMC becomes a Gaussian mixture model (GMM) that exclusively clusters peptides according to their phosphorylation signal. With a very large weight, DDMC primarily clusters peptides according to their peptide sequences. Clustering both the sequence and abundance measurements ensures that the resulting clusters are a function of both features, which we hypothesized would provide both more meaningful and robust clusters.

The resulting clustering provides coordinated outputs that can be used in a few different ways. The cluster centers, by virtue of being a summary for the abundance changes of these peptides, can be regressed against phenotypic responses (e.g., cell phenotypes or clinical outcomes) to establish associations between clusters and response (Figure 1B). Regression using the clusters instead of each peptide ensures that the model can be developed despite relatively few samples, with minimal loss of information since each peptide within a cluster varies in a similar manner. One can also interrogate the position-specific scoring matrices (PSSMs) from the resulting cluster sequence motifs. Given a set of peptide sequences, PSSMs quantify the amino acid frequencies across peptide positions and show to what extent each residue is enriched or depleted per position (Figure 1A). Thus, a cluster PSSM provides a general representation of the cluster sequence features and can be readily compared with other information, such as experimentally generated profiles of putative upstream kinases via position-specific scanning libraries (PSPLs) (Obata et al., 2000; Snyder et al., 2010). In this technique, a kinase of interest is individually incubated with each of 180 different peptide libraries in which each library contains a central phosphoacceptor residue (S/T or Y), a second fixed amino acid located any of the peptide residues spanning positions −5 throughout +4 relative to the phosphorylation site, and a degenerate mixture containing all natural amino acids at all other positions. The kinase and peptide libraries are incubated in the presence of radioactive ATP, which allows the quantification of phosphorylation abundance per residue and position and the identification of the kinase’s “optimal” substrate motif. We extracted a collection of 42 kinase specificity profiles to identify which cluster motifs most resemble the optimal motif of putative upstream kinases (Figure 1C) (Hutti et al., 2004; Miller et al., 2008; Begley et al., 2015; van de Kooij et al., 2019). However, as kinase-substrate specificity is also dictated by features outside of the immediate substrate region, we also note that our approach is more general than strictly assembling kinase-substrate predictions, as non-enzymatic specificity information may be present in the DDMC sequence motifs. This overview demonstrates how DDMC can take complex, coordinated signaling measurements and find patterns in the phosphorylation signals to reconstruct signaling networks and associate clusters and phenotypes.

### DDMC robustly imputes missing values

**Figure 2.**
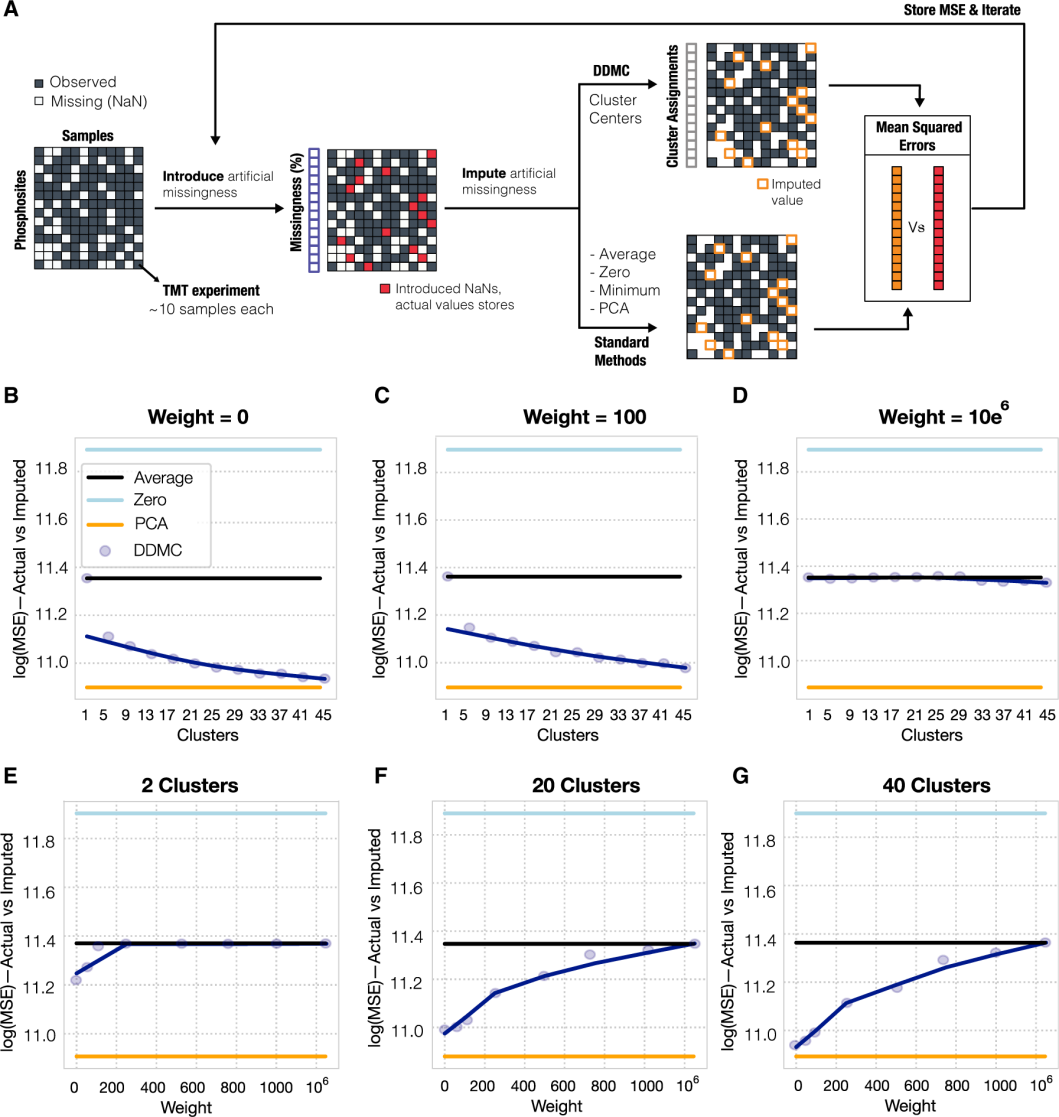
Benchmarking the robustness of motif clustering to missing measurements (A) A schematic of the process for quantifying robustness to missing values. Any peptides containing fewer than seven TMT experiments were discarded. For the remaining 15,904 peptides, an entire random TMT experiment was removed per peptide and these values were stored for later comparison. Next, these artificial missing values were imputed using either a baseline strategy (peptide mean signal, constant zero, or matrix completion by PCA with five components) or the corresponding cluster center. Once a MSE was computed for each peptide, the second iteration repeats this process by removing a second TMT experiment. (B–G) A total of five random TMT experiments per peptide were imputed by clustering using a different number of clusters (B–D) or different weights (E–G). Note that the minimum signal imputation is not shown for clarity since its prediction performance was dramatically worse.

A major limitation of discovery-mode MS-based phosphoproteomic data is the presence of missing values due to the stochastic signaling coverage in each run. In the resulting dataset, many phosphosites are observed in groups of samples and missed in others (Figure 2A). To evaluate the robustness of DDMC in analyzing incomplete datasets, we designed a computational experiment wherein we synthetically removed random TMT experiments from the dataset and predicted them using the peptide-assigned cluster centers. The mean squared error of imputation was compared with other commonly used strategies, such as the peptides’ mean, filling in zeros, or matrix completion by principal-component analysis (PCA) (Figure 2A). We applied this experiment across different numbers of clusters and sequence weighting to explore the imputation performance. We observed that increasing the number of clusters consistently improved performance (Figures 2B and 2C), whereas primarily prioritizing the sequence information yielded worse imputation estimates (Figures 2D–2G). However, a weight of 100 still allowed DDMC to accurately predict missing values while incorporating the sequence information into the clustering criterion (Figures 2C and 2E–2G). We concluded that DDMC clearly outperforms many common imputation strategies and imputes missing values with similar accuracy to matrix completion by PCA.

### DDMC correctly identifies AKT1 and ERK2 as upstream kinases of signaling clusters containing their substrates

**Figure 3.**
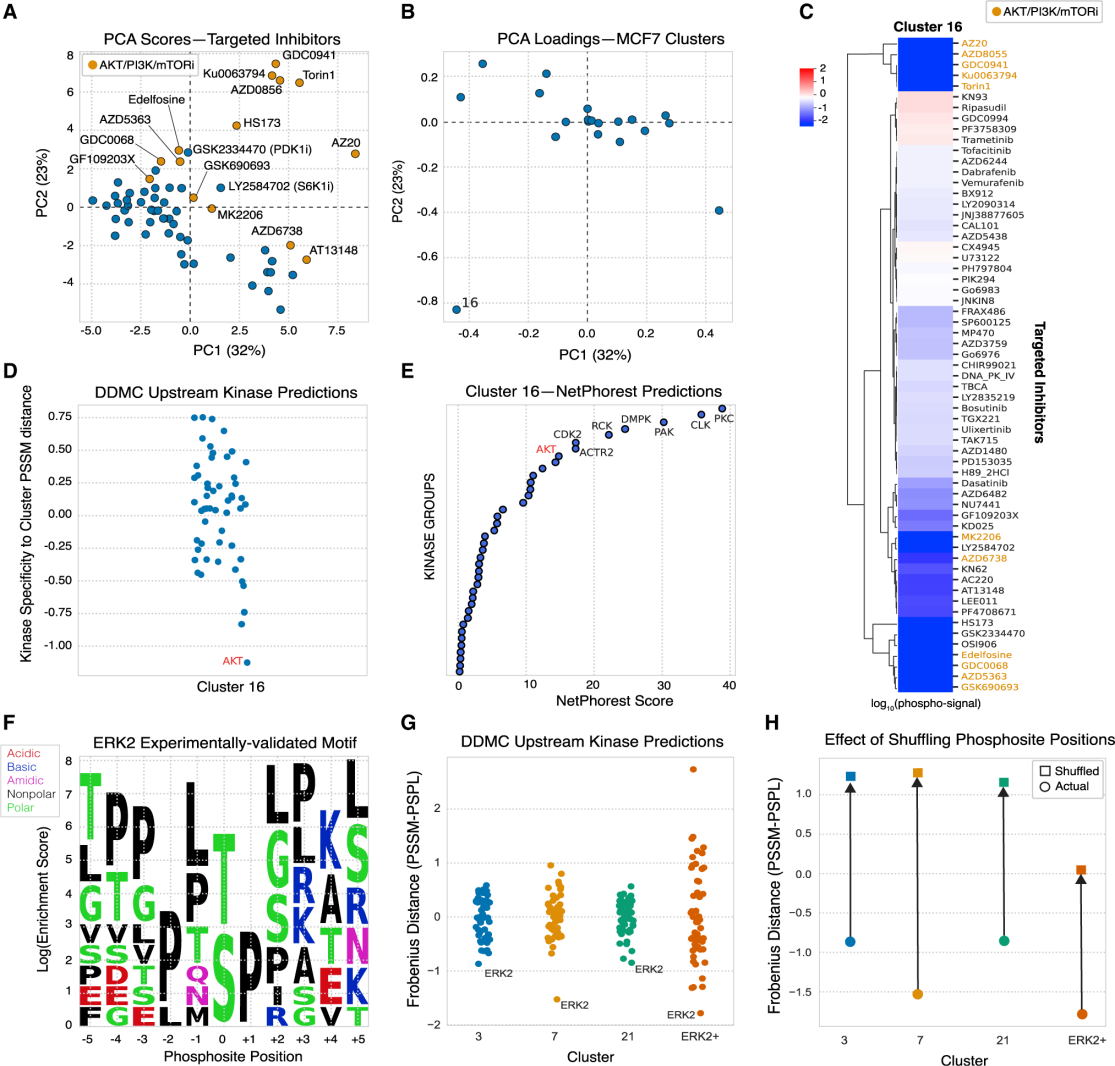
Validation of upstream kinase predictions (A and B) PCA analysis of the DDMC phosphoproteome clusters of MCF7 cells subjected to a drug screen (Hijazi et al., 2020). (C) Heatmap showing the effect of inhibitors on the phosphorylation signal of cluster 16. (D) DDMC upstream kinase prediction of cluster 16. (E) NetPhorest upstream kinase prediction of cluster 16. (F) Resulting PSSM generated using reported ERK2 substrates (Carlson et al., 2011). (G) Upstream kinase predictions of CPTAC clusters 3, 7, and 21 in addition to the ERK2 motif shown in (F). (H) Upstream kinase predictions of the same PSSMs after randomly shuffling the motif positions.

A major benefit of directly modeling the phosphopeptide sequence information is the construction of cluster motifs to infer which putative upstream kinases might preferentially target a specific cluster. To validate this ability, we used DDMC to cluster the phosphoproteomic measurements of MCF7 cells treated with a panel of 61 drug inhibitors reported by Hijazi et al. (2020). We hypothesized that the phosphoproteomic clusters align to specific and identifiable targeted kinases. Examining the clusters by PCA, the scores of AKT/PI3K/mTOR targeted inhibitors (shown in orange in Figure 3A) and the loading of cluster 16 were clearly opposed (Figures 3A and 3B). The additional inhibitors GSK2334470 and LY2584702 were also negatively associated with cluster 1; both inhibit kinases PDK1 and S6K1, respectively, expected to modulate the AKT/PI3K/mTORC pathway. A heatmap displaying cluster 1’s phosphorylation signal across treatments corroborates that the abundance of these peptides is substantially decreased when treated with AKT/mTOR/PI3K inhibitors (Figure 3C). Encouragingly, the AKT profile was most closely matched to the PSSM of cluster 1 within a collection of 42 different kinase PSPL matrices (Figure 3D). In addition, NetPhorest identified AKT as the eighth top scoring upstream kinase of cluster 1, further corroborating DDMC’s prediction (Figure 3E).

As a second test, we extracted the sequences of experimentally validated substrates of ERK2 to create an “artificial” ERK2-specific PSSM positive control (ERK2+ motif) (Carlson et al., 2011) (Figure 3F). As expected, ERK2 was predicted to be the upstream kinase with the highest preference for the cluster’s motif (Figure 3G). Given the consistent enrichment of hydrophobic and polar residues throughout the entire ERK2 target motif (Figure 3F), we asked whether randomly shuffling the cluster PSSM positions surrounding the phosphoacceptor residue would affect the upstream kinase prediction. Randomization led to a marked increase in the distance between the ERK2 specificity profile and the ERK2+ motif (Figure 3H). Clusters from the CPTAC dataset that were preferentially favored by ERK2 showed a similar decline in specificity between the clusters PSSMs and ERK2 PSPL matrix on randomization (Figure 3H). This experiment shows that position-specific matching information is contained within the ERK2 target motif despite the uniform biophysical properties (Figures 3G and 3H). Altogether, these results illustrate two different validation scenarios in which DDMC successfully identifies the upstream kinases regulating clusters.

### DDMC improves prediction of different phenotypes and provides more robust clustering

As detailed later (Figures 5, 6, and 7), we used DDMC to analyze the phosphoproteomes of 110 treatment-naïve LUAD tumors and 101 paired NATs from the NCI’s CPTAC LUAD study. We used DDMC with the binomial sequence distance method and 30 clusters (Figures 1 and 2B–2D). We were able to include 30,561 peptides that were not observed in every sample through our ability to handle missing data but still filtered out 11,822 peptides that were only captured in one 10-plex TMT run. We used this fitting result throughout the rest of this study. The resulting cluster motifs can be found in Figure S2.

**Figure 4.**
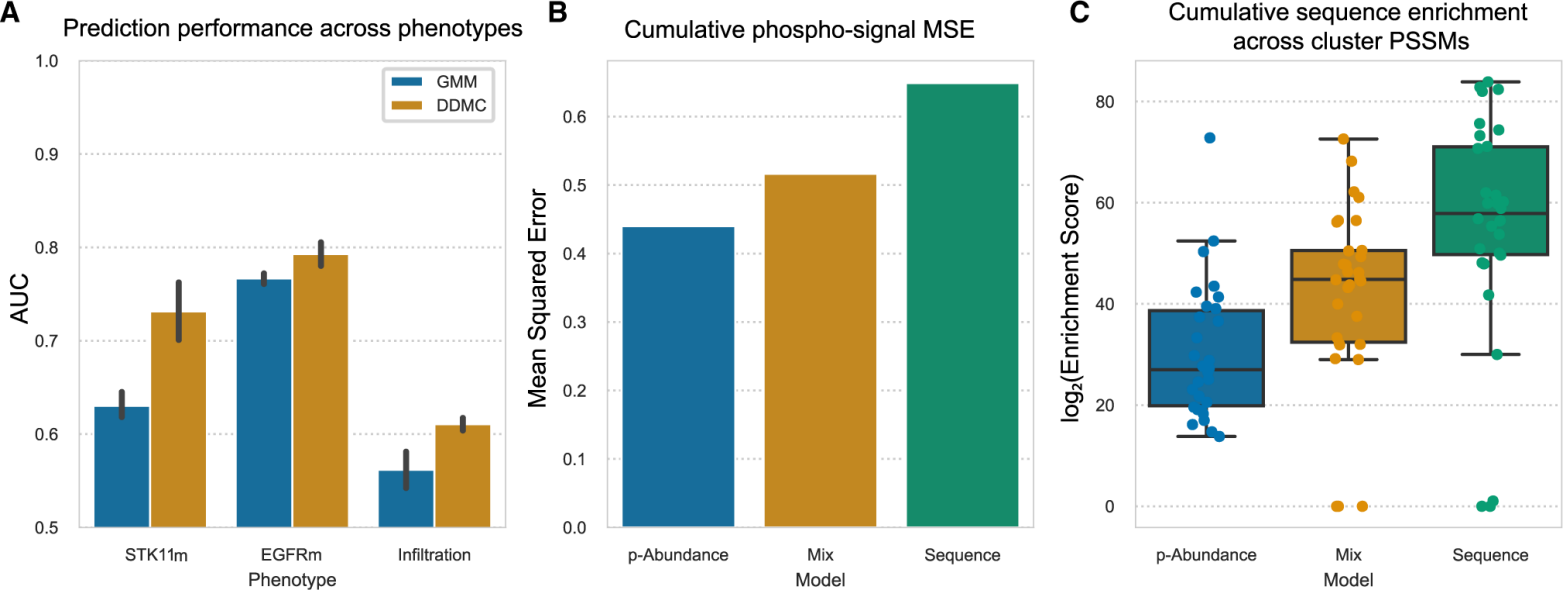
Sequence information enhances model prediction and provides more robust clustering (A) Performance of a regression model predicting the mutational status of STK11 and EGFR, and the level of tumor infiltration in LUAD patients using either only phosphorylation abundance (weight = 0), mainly sequence information (10^6^), or both (0 < w ≤ 10^6^). Error bars indicate the standard error of the mean. (B) MSE between the phosphorylation signal of 2,000 randomly selected peptides and the center of its assigned clusters using a weight of 0 (p-Abundance), 250 (Mix), or 10^6^ (Sequence). (C) Cumulative PSSM enrichment across positions comparing the p-Abundance, Mix, and Sequence clustering strategies. Error bars indicate the 95% confidence interval. The bottom and top of the box indicate the 25th and 75th percentiles. The line inside the box is the median. See also Figure S3

To evaluate the benefit of including peptide sequence information during clustering, we investigated whether different sequence weights would affect the performance of a regularized logistic regression model that predicts the mutational status of STK11, whether a patient harbors a mutation in the epidermal growth factor receptor (EGFRm), and the level of tumor infiltration (“hot” versus “cold”). Three independent DDMC runs were performed to observe the reproducibility of the prediction results. We found that for all three phenotypes, optimal predictions were derived when clustering was partly based on the peptide sequence—as highlighted in red circles. In the case of STK11, the use of the maximum performance is achieved with a weight of 250. Likewise, EGFRm samples were best classified with a mix weight of 1,000. Finally, the regression model classifying whether a sample is “hot-tumor-enriched” (HTE) or “cold-tumor-enriched” (CTE) showed the best fitness with weights spanning from 100 to 750. Together, these results indicate that observing the motif information during clustering leads to final clusters that enhance the performance of downstream phenotype prediction models (Figures 4A and S3). Note that random chance is equal to 0.5 and perfect predictions 1.0, so an improvement of 0.1 (STK11 prediction) is a movement across 20% of this range.

Next, we explored how using different weights affects the overall phosphorylation signal and sequence information of the resulting clusters. To do so, we compared the model behavior after clustering the CPTAC data with a weight of 0 (peptide abundance only), 100 (mix), and 1,000,000 (mainly sequence). First, we hypothesized that the abundance-only model would generate clusters wherein its members would show less intra-cluster variation in phosphorylation signal and thus a lower mean squared error (MSE). To test this, we computed the average peptide-to-cluster MSE of 2,000 randomly selected peptides for each model across all clusters. We observed a direct correlation between weight and MSE (Figure 4B). Next, we calculated the cumulative PSSM enrichment by summing the sequence information (bits) of all cluster PSSMs per model. As expected, increasing the weight led to a corresponding increase in the cumulative sequence information (Figure 4C). We additionally observed that the clustering results generated by DDMC are noticeably different from those of eight standard clustering methods (Figure S4).

We compared the classification performance of four regularized logistic regression models fit either the DDMC clusters, clusters generated by the standard methods GMM and k-means, or the raw phosphoproteomic data directly. It is worth noting that unlike DDMC, methods such as GMM, k-means, or direct regression cannot handle missing values. and thus for these strategies we used the 1,311 peptides that were observed in all samples, whereas DDMC was fit to the entire dataset comprising 30,561 phosphosites. In predicting STK11 mutational status, we found that DDMC fit to the fully observed 1,311 peptides yielded a moderately higher prediction performance than k-means, GMM, and DDMC fit to the entire dataset with missingness (Figure S3A). EGFR mutational status was noticeably better classified with both DDMC fittings (with and without missingness) than with k-means and GMM. Direct regression to the raw signaling data yielded excellent performance; however, this strategy assigns thousands of coefficients to different peptides that vary every time the model is run, rendering this approach unable to establish a consistent link between phenotypes and signaling (Figure S5D). These results show that using DDMC with a mixed weight that similarly prioritizes both information sources—peptide abundance and sequence—leads to more robust clustering of phosphosites through a tradeoff between phosphorylation abundance and sequence motifs.

### Widespread, dramatic signaling differences exist between tumor and normal adjacent tissue

**Figure 5.**
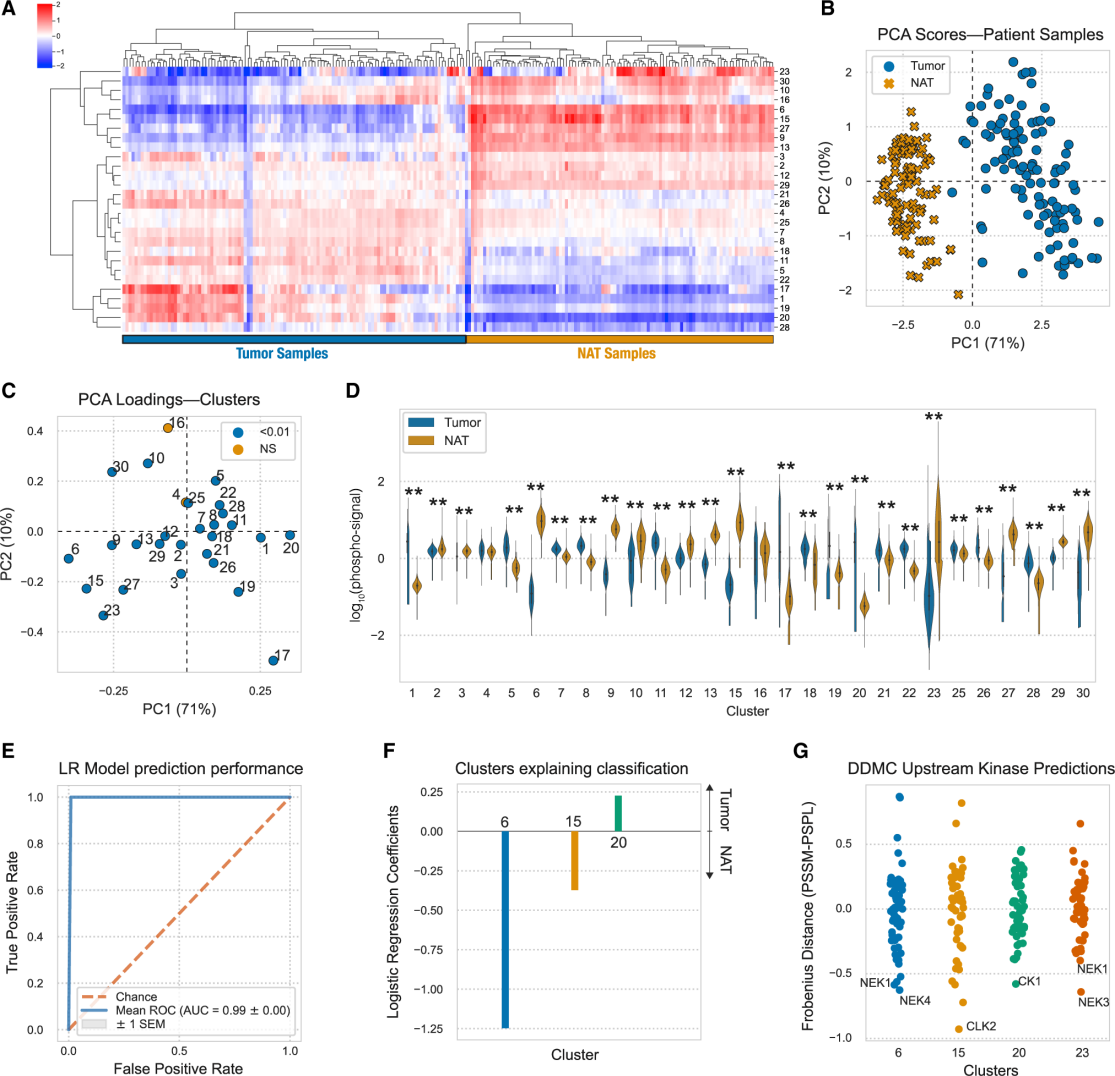
Conserved tumor differences compared with normal adjacent tissue (A) Hierarchical clustering of the DDMC cluster centers. (B and C) PCA scores (B) and loadings (C) of the samples and phosphopeptide clusters, respectively. (D) Phosphorylation signal of tumor and NAT samples per cluster and statistical significance according to a Mann-Whitney *U* rank test (*p < 0.05; **p < 0.001). (E) Receiver operating characteristic curve (ROC) of a regularized logistic regression model. (F) Logistic regression weights per cluster. (G) Upstream kinase predictions of clusters 6, 15, 20, and 23. See also Figure S5

We explored whether DDMC could recognize conserved signaling patterns in tumors compared with NAT. The signaling difference between tumors and NAT samples was substantial, highlighting the significant signaling rewiring in tumor cells (Figure 5A). Using PCA, we could observe that NAT samples were more like one another than to each tumor sample (Figures 5B and 5C). Nearly every cluster was significantly different in its average abundance between tumor and NAT (Figures 5C and 5D). Not surprisingly given these enormous differences, samples could be almost perfectly classified using their phosphopeptide signatures, with or without DDMC (Figures 5E and S5A–S5C). However, directly classifying samples using the unclustered phosphoproteomic data and a regularized logistic regression model generates phosphosite weights that vary across runs. For instance, we saw that the associations of peptides MYH9: S1943-p, IFT140: S1443-p, and NCK1: Y105-p were selected in two runs but had an opposite association with sample status (Figure S5D). Using the DDMC clusters, a logistic regression model identified consistent associations between NAT versus tumor status and clusters 6, 15, and 20 (Figures 5E and 5F). With the abundance changes and regression results we observed, we further explored these three clusters.

Our DDMC results suggest that downregulation of NEKs and CLK2 promote cilia disassembly and migration in cancer cells, respectively, while CK1 activity correlates with tumor-specific signaling regulating cell cycle. Peptides in cluster 6, presumably targeted by NEK1&4, associate with hepatocyte growth factor (HGF) receptor signaling as well as cytoskeletal remodeling phenotypes (Figures 5G and S6A). Even though NEKs are fairly understudied, NEK1 has an established role in ciliagenesis and NEK4 is involved in regulating microtubule dynamics (Moniz et al., 2011; Meirelles et al., 2014). The absence of cilia in cancer cells promotes malignancy (Plotnikova et al., 2008; Fabbri et al., 2019), and NEK-regulated cluster 6 displays a striking phosphorylation decrease in tumor samples compared with NATs, which might result in cilia disassembly. Interestingly, cluster 23, also downregulated in tumors, presents a motif favored by NEK1&3 and shows a marked enrichment of cilia-related processes (Figures 5D and S6A).

Similarly, cluster 15 is dramatically upregulated in NAT versus tumor samples, contributes toward correctly classifying NAT samples, and DDMC predicts CLK2 to be the most promising candidate for regulating its activation. CLK2 is a largely understudied dual specificity kinase known to act as an RNA splicing regulator. Gene set enrichment analysis (GSEA) indicates that integrin-mediated cell adhesion, cell junction assembly, and organization are the biological processes with highest enrichment scores (Figure S6A and S6B). These data are consistent with the observation that CLK2 downregulation enhanced cell migration and invasion and upregulated epithelial-to-mesenchymal transition (EMT)-related genes (Yoshida et al., 2015).

Conversely, the phosphorylation signal in cluster 20 is significantly higher in tumors compared with NATs and explains tumor-specific signaling that could be driven by CK1 (Figures 5D and 5F). CK1 has been identified to induce acquired resistance to the EGFR inhibitor erlotinib in several EGFR-mutant non-small cell lung cancer (NSCLC) cell lines (Lantermann et al., 2015). Taken together, DDMC builds phosphoproteomic clusters that present signaling dysregulation common to tumors compared with NATs and identifies putative upstream kinases modulating them.

### Genetic driver mutations are associated with more targeted phosphoproteomic rewiring

Tyrosine kinase inhibitors (TKIs) targeting the receptor tyrosine kinase (RTK) EGFR are effective treatments in cancer patients with EGFRm. However, these treatments are limited by drug resistance, which in some cases is mediated by cell signaling rewiring that bypasses EGFR inhibition. Thus, we aimed to identify the phosphoproteomic aberrations triggered by mutant EGFR.

**Figure 6.**
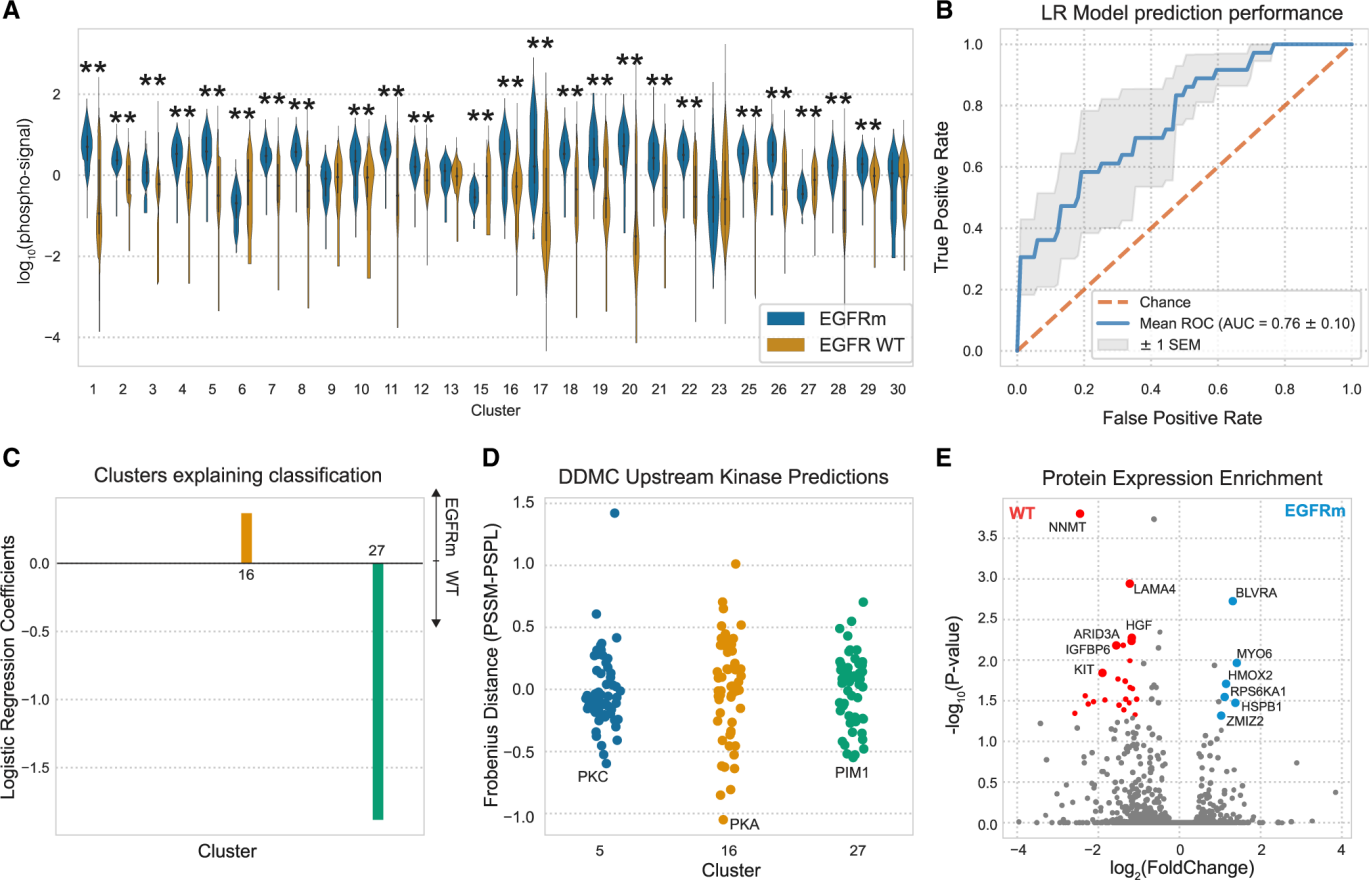
Phosphoproteomic aberrations associated with EGFR mutational status (A) Phosphorylation signal of EGFR WT and mutant samples per cluster and statistical significance according to a Mann-Whitney *U* rank test (*p < 0.05; **p < 0.001). (B and C) ROC of a logistic regression model predicting the EGFR mutational status and (C) its corresponding weights per sample type. (D) Putative upstream kinases of clusters 5, 16, and 27. (E) Volcano plot showing the differential protein expression between EGFR WT and mutant samples. Colored dots are statistically significant according to a Mann-Whitney *U* rank test (p values < 0.05).

Most clusters were significantly altered on average, generally toward higher abundances with an EGFR mutation (Figure 6A). The cluster centers corresponding to each patient’s tumor samples, excluding NATs, could successfully predict the EGFR mutational status by regularized logistic regression. We observed the largest statistically significant phosphorylation abundance increase in EGFRm samples with cluster 5 (Figure 6B). Moreover, the regression model identified clusters 16 and 27 to explain the signaling differences between EGFRm and wild-type (WT) samples, respectively (Figure 6C). DDMC identified PKC, PKA, and PIM1, respectively, as putative upstream kinases of clusters 5, 16, and 27 (Figure 6D). As elaborated below, our data suggest that EGFRm tumors might be regulated by two groups of proteins acting downstream of PKC and PKA, whereas PIM1 might support the signaling of EGFR WT tumors that are possibly driven by further RTKs.

In different EGFR-dependent tumors, PKC—putative regulator of cluster 5—has been shown to mediate receptor transactivation, induce mTOR signaling, and confer acquired resistance to EGFR inhibitors (Stewart and O’Brian, 2005; Fan et al., 2009; Salama et al., 2016; Chen et al., 2021). Enrichment analysis of the global protein expression data across all tumor samples showed that the heme degradation pathway enzymes BLVRA and HMOX2, as well as the mitogenic kinase RPS6KA1, among others, are significantly upregulated in EGFRm samples (Figure 6E). Consistent with the DDMC prediction, the kinase domains of RPS6KA1 and BVLRA are phosphorylated by PKC (Meshki et al., 2010; Miralem et al., 2012). GSEA shows an over-representation of the EGFR, human epidermal growth factor receptor (HER), and vascular endothelial growth factor receptor (VEGFR) signaling pathways in cluster 5, which might suggest crosstalk among the three RTKs’ signaling (Figure S6A).

PKA, which might regulate cluster 16, is crucial for EMT, migration and invasion, and tumorigenesis (Shaikh et al., 2012; Coles et al., 2020). This kinase induces the activation of EGFR and its inhibition leads to a ligand-independent degradation of the receptor (Chen et al., 2002; Piiper et al., 2003; Oksvold et al., 2008; Feng et al., 2014). The EGFR and VEGFR signaling pathways are also enriched in cluster 16 alongside the ATM pathway (Figure S6A).

PIM1 might act upstream of cluster 27, which in turn is upregulated in EGFR WT tumor samples (Figures 6A, 6C, and 6D). PIM1 is an established oncogenic driver, and its inhibition was shown to re-sensitize cancer cells to radiotherapy as well as c-MET and ALK inhibition in NSCLC tumors (Kim et al., 2013; Cao et al., 2019; Trigg et al., 2019; Attili et al., 2020). Interestingly, the c-MET ligand HGF is more abundant in EGFR WT samples (Figure 6E). Moreover, activation of the KIT receptor, which can also mediate bypass resistance to targeted therapies and is enriched in EGFR WT samples, is reportedly regulated, at least in part, by PIM1 (An et al., 2016; Dziadziuszko et al., 2016; Ebeid et al., 2020) (Figures 6D and 6E). In total, our analysis identifies a consistent association between EGFR activity with established and previously unknown signaling mechanisms.

Finally, to show that DDMC can accurately predict other genotypes, we again used the signaling cluster centers with regularized logistic regression to classify the mutational status of STK11. Inactivating somatic mutations in the tumor suppressor STK11 leads to increased tumorigenesis and metastasis (Ji et al., 2007). This context is consistent with our results that clusters 9 (TLK1) and 11 (CK2) are associated with STK11m signaling, whereas clusters 16 (PKA) and 18 (CK1) are associated with WT samples (Figure S7).

### Exploration of immune infiltration-associated signaling patterns in tumors

Immune checkpoint inhibitors (ICIs) have emerged as effective treatment options for NSCLC patients. However, there still is a need to identify or influence which patients will respond to these therapies. Patients who do not respond to ICIs often have tumors with poor immune infiltration either inherently or via an adaptive process after long exposure to the drug. However, the signaling mechanism by which malignant cells prevent tumor infiltration remains elusive. We used our DDMC clusters to explore the shared signaling patterns that differentiate HTE from CTE LUAD patients. HTE and CTE status per patient was determined using xCell (Aran et al., 2017; Gillette et al., 2020).

**Figure 7.**
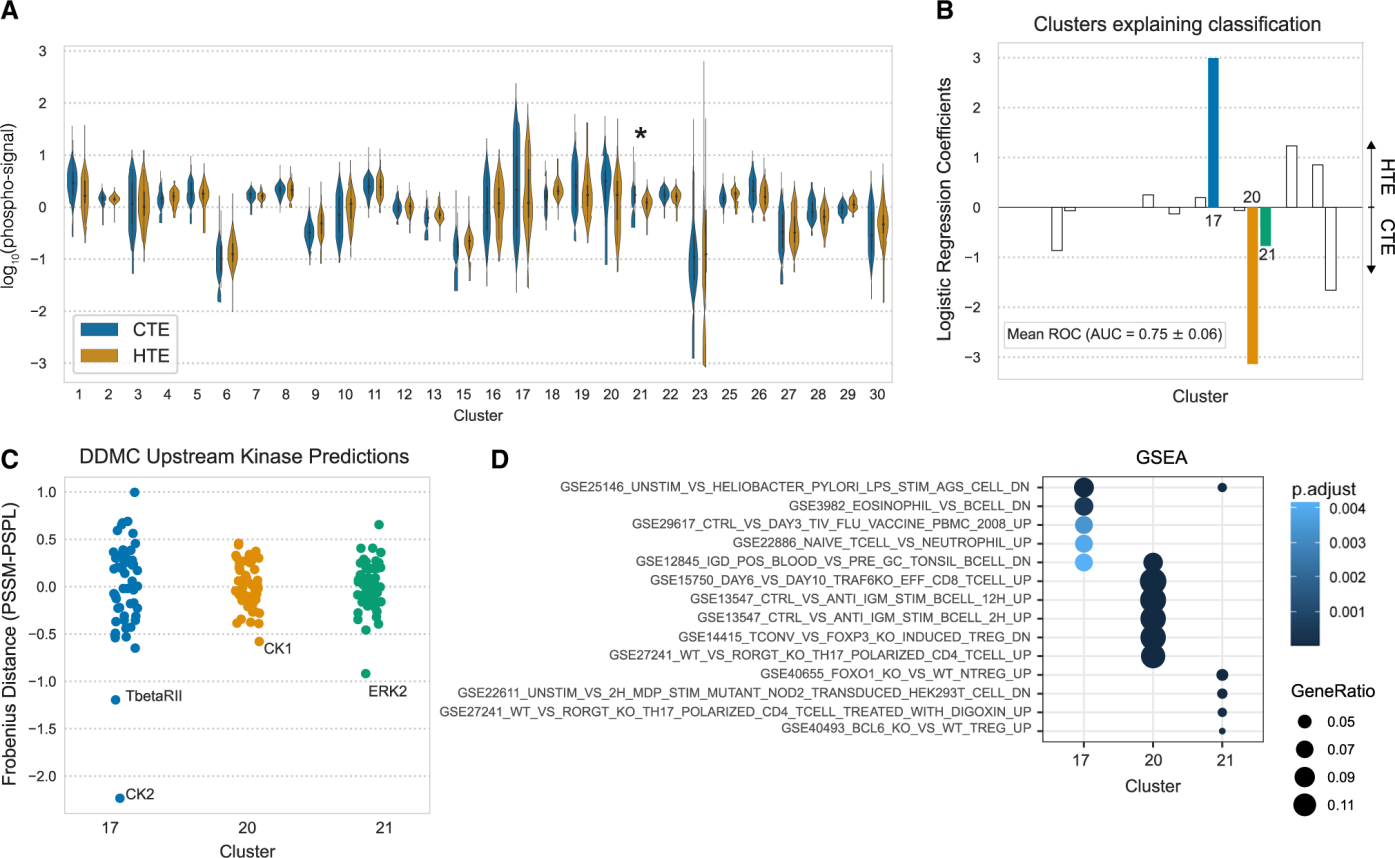
Phosphoproteomic signatures correlating with tumor immune infiltration (A) Phosphorylation abundance of CTE and HTE samples per cluster and statistical significance according to a Mann-Whitney *U* rank test (*p < 0.05; **p < 0.001). (B) Mean ROC and coefficients of a logistic regression model predicting infiltration status—cold-tumor enriched (CTE) versus hot-tumor enriched (HTE). (C) Putative upstream kinases of clusters 17, 20, and 21. (D) GSEA of immunological processes.

Only cluster 21 had a significantly different abundance between CTE and HTE samples (Figure 7A); however, infiltration status could still be accurately classified using combinations of the DDMC clusters. This predictive performance was mainly explained by a positive association of cluster 17 with HTE status and clusters 20 and 21 with CTE samples. Other clusters contributed to explain the signaling differences between both groups but to a lesser extent (Figure 7B). These results prompted us to further investigate clusters 17, 20, and 21, which our model inferred were regulated by CK2/TGFBR2, CK1, and ERK2, respectively (Figure 7C). We found that CK2 and TFGBR2 associate with the regulation of B cell homeostasis in HTE samples, whereas CK1 and ERK2 correlate with the activity of immunosuppressive regulatory T cells (Tregs) in CTE samples.

We performed GSEA on these three clusters using a compendium of gene sets associated with immunological signatures (Godec et al., 2016). Cluster 17 presents a marked enrichment of downregulated genes upon lipopolysaccharide stimulation, an upregulation of B cell- over eosinophil-specific genes, the enrichment of genes upregulated by an influenza vaccine, and genes upregulated in immunoglobulin (Ig)D+ B cells. Thus, these might suggest that CK2 and TGFBR2 could regulate cluster 17 to direct B cell homeostasis. In line with this interpretation, a recent study found that CK2 knockout in B cells resulted in lower B cell receptor signaling, which perturbed B cell differentiation (Wei et al., 2021). Transforming growth factor (TGF)-β signaling is involved in several processes regulating B cell maturation. For instance, a study showed that IgD+ B cells were observed in the presence of TGF-β signaling, whereas genetic deletion of the receptor led to complete loss of IgD (Albright et al., 2019).

Consistent with their higher abundance in CTE samples and negative logistic regression coefficients, both cluster 20 and 21 showed enrichment of several phenotypes describing the induction of Tregs. ERK2 is known to modulate PD-L1 expression and its inhibition has been shown to improve anti-PD-L1 blockade in several cancer types, including NSCLC (Ng et al., 2018; Kumar et al., 2020; Henry et al., 2021; Luo et al., 2021). Conversely, while CK1 is associated with tumorigenesis, tumor growth, and drug resistance in cancer cells, its role in different immune cells and its ability to promote immune evasion has not been addressed. Overall, these data demonstrate that the presence or lack of tumor immune infiltration can be accurately predicted by the DDMC clusters, which in turn help identify putative upstream kinases modulating immune evasion.

## DISCUSSION

Phosphorylation-based cell signaling through the coordinated activity of protein kinases allows cells to swiftly integrate environmental cues and orchestrate a myriad of biological processes. MS-based global phosphoproteomic data provide the unique opportunity to globally interrogate signaling networks to better understand cellular decision-making and its therapeutic implications. However, these data also present challenging issues because of their incomplete and stochastic coverage, high-content but low-sample throughput, and variation in coverage across experiments. Here, we propose a clustering method, DDMC, that untangles the coordinated signaling changes by grouping phosphopeptides based on their phosphorylation behavior and sequence similarity (Figure 1). To test the utility of DDMC, we clustered the phosphoproteomes of LUAD patients and used the resulting groups of peptides to decipher signaling dysregulation associated with tumors, genetic backgrounds, and tumor infiltration status (Figures 5, 6, and 7).

Previous efforts in regressing MS-based phosphorylation measurements against phenotypic or clinical data have been based on the ability of certain regression models such as PLSR or LASSO to robustly predict using high-dimensional and correlated data (Kourou et al., 2015). While these models can generally be predictive with such data, they are not easily interpretable (Figure S5D). We hypothesized that clustering large-scale MS measurements based on biologically meaningful features and using the cluster centers could enhance the predictive performance of the model while providing highly interpretable results, wherein clusters constitute signaling nodes distinctly correlated with patient phenotypes. Here, we demonstrate that DDMC enhances model prediction and interpretation (Figures 3, 4A, and S3).

A key benefit of DDMC is that the identified clusters are not limited to pre-existing motifs and are therefore not dependent on prior experimentally validated kinase-substrate interactions. This method could therefore likely improve our understanding of the signaling effects of understudied kinases. For instance, our model predicts that NEKs promote, at least in part, a cluster with strikingly increased signaling in NATs compared with tumors. Further exploration of this cluster led us to hypothesize that the lack of NEK signaling in tumor samples might be associated with the absence of cilia in lung tumors (Figures 5G and S6A). In addition, we show that cluster 20 greatly contributes to explain a low immune infiltration status and might be regulated by the kinase CK1, which to our knowledge has not been studied in this context. While DDMC models the peptide sequence information without any constraints or assumptions defined by prior knowledge, the method could be easily adapted to populate clusters with the substrate motif information of specific upstream kinases. This “fixed” method could help improve granularity within a specific kinase signaling pathways.

An additional major challenge during the analysis of large-scale signaling data is missingness. Statistical tools often require complete datasets and, while researchers can use standard methods to impute missing values such as the peptides’ mean signal, imputation strategies generally work best when missing values only comprise a small fraction of the dataset (Chen et al., 2020; Deb et al., 2020; Gillette et al., 2020). In this study we show that DDMC can model a dataset of 30,561 peptides after filtering out any phosphosites that were not captured in more than one TMT run (up to ~80% of missingness) by imputation during the expectation-maximization (EM) fitting process (see STAR Methods). Furthermore, DDMC clearly outperforms the imputation performance of using the peptides’ mean or constant zero and provides similar results to PCA imputation (Figure 3). This important feature could offer the possibility of conducting pan-cancer phosphoproteomics studies using readily available large-scale clinical phosphoproteomic data by overcoming the fractional overlap in peptide coverage.

More generally, DDMC is tailored to model any biological datasets that combine a given signal with sequence information. In addition to TMT multiplex liquid chromatography-tandem MS datasets (as used here), this method may be equally useful with other techniques such as targeted MS via data-independent acquisition (Venable et al., 2004; Gillet et al., 2012). Beyond phosphoproteomics, DDMC can also be used to cluster transcription factor motifs or neoantigen sequences coupled with their gene or protein expression data. The benefit of building algorithms combining different information sources is evident in previously published approaches. For instance, INKA predicts active kinases by integrating scores reflecting both phosphorylation status and substrate abundance (Beekhof et al., 2019). A similar approach to that taken here could be applied with other generative algorithms, such as probabilistic PCA or probabilistic generative adversarial networks, with similar benefits. Integrating yet other information may reveal further improvements in the dimensionality reduction and interpretation of other high-throughput molecular measurements.

In total, we show that combining the information about the sequence features and phosphorylation abundance leads to more robust clustering of global signaling measurements. Use of the DDMC clusters to regress against cell phenotypes led to enhanced model predictions and interpretation. Thus, we propose DDMC as a general and flexible strategy for phosphoproteomic analysis.

### Limitations of this study

Our present analysis is limited to a single clinical phosphoproteomics dataset. Examining other datasets, and integrating phosphoproteomics measurements with other molecular measurement modalities, will reveal new insights and other ways to improve the model. For instance, it remains unclear how DDMC might perform with smaller cohorts or with measurements across different cancer types.

DDMC interpretation is enhanced by comparing the resulting cluster PSSMs with kinase specificity data such as PSPL to identify putative upstream kinases for each cluster. Validation experiments showed that DDMC was able to correctly associate AKT1 and ERK2 with clusters containing their respective substrates (Figure 3). Kinase specificity is defined by additional features beyond the phosphosite motif, however, such as kinase-substrate co-localization, regulation by phosphosite-binding domains (e.g., SH2, PTB domains), or docking. These other kinase regulatory processes could compromise kinase-cluster associations established by DDMC. Refined methods of quantifying kinase specificity, alongside adjustments to DDMC to account for these other regulatory processes, could improve both upstream kinase predictions and the resulting peptide clustering (Shah et al., 2018).

## STAR★METHODS

### RESOURCE AVAILABILITY

#### Lead contact

Further information and requests for resources should be directed to and will be fulfilled by the lead contact, Aaron Meyer (ameyer@asmlab.org).

#### Materials availability

This study did not generate new unique reagents.

#### Data and code availability

- No new standardized datasets were generated by this study.
- All original code has been deposited at Zenodo and is publicly available as of the date of publication. The DOI is listed in the key resources table.
- Any additional information required to reanalyze the data reported in this paper is available from the lead contact upon request.

### METHOD DETAILS

#### Expectation-maximization (EM) algorithm architecture

We constructed a modified mixture model that clusters peptides based on both their abundance across conditions and sequence. The model is defined by a given number of clusters and weighting factor to prioritize either the data or the sequence information. Fitting was performed using expectation-maximization, initialized at a starting point. The starting point was derived from k-means clustering the abundance data after missing values were imputed. During the expectation (E) step, the algorithm calculates the probability of each peptide being assigned to each cluster. In the maximization (M) step, each cluster’s distributions are fit using the weighted cluster assignments. The peptide sequence and abundance assignments within the E step are combined by taking the sum of the log-likelihood of both assignments. The peptide log-likelihood is multiplied by the user-defined weighting factor immediately before to influence its importance. Both steps repeat until convergence as defined by the increase in model log-likelihood between iterations falling below a user-defined threshold.

#### Phosphorylation site abundance clustering in the presence of missing values

We modeled the log-transformed abundance of each phosphopeptide as following a multivariate Gaussian distribution with diagonal co-variance matrix. Each dimension of this distribution represents the abundance of that peptide within a given sample. For example, within a data set of 100 patients and 1000 peptides, using 10 clusters, the data is represented by 10 Gaussian distributions of 100 dimensions. Unobserved/missing values were initially indicated as NaN and subsequently imputed using the method SoftImpute (using the package fancyimpute) upon model initialization. During every iteration of the EM algorithm, the missing values are then updated according to the current model. Any peptides that were detected in only one TMT experiment were discarded.

#### Sequence-cluster comparison

##### PAM250

During model initialization, the pairwise distance between all peptides in the dataset was calculated using the PAM250 matrix. The mean distance from each peptide to a given cluster could then be calculated by:

$$w=\frac{1}{n}(P\cdot v)$$

where *P* is the *n* × *n* distance matrix, *n* is the number of peptides in the dataset, *v* is the probability of each peptide being assigned to the cluster of interest, and *w* is the log-probabilities of cluster assignment.

##### Binomial enrichment

We alternatively used a binomial enrichment model for the sequence representation of a cluster based on earlier work (Schwartz and Gygi, 2005). Upon model initialization, a background matrix *i* × *j* × *k* was created with a position-specific scoring matrix of all the sequences together. Next, a data tensor *T*T was created where *i* is the number of peptides, *j* is the number of amino acid possibilities, and *k* is the position relative to the phosphorylation site. This tensor contained 1 where an amino acid was present for that position and peptide, and 0 elsewhere.

Within each iteration, the cluster motif would be updated using *v*, the probability of each peptide being assigned to the cluster of interest. First, a weighted count for each amino acid and position would be assembled:

$$k={\left(T^{⊤}\cdot v\right)}^{⊤}$$

Because peptides can be partially assigned to a cluster, the counts of each amino acid and position can take continuous values. We therefore generalized the binomial distribution to allow continuous values using the regularized incomplete Beta function:

$$M=B\left({\left\|\vec{v}\right\|}_{1}-k,k+1,1-G\right)$$

Finally, the log-probability of membership for each peptide was calculated based on the product of each amino acid-position probability.


w=log(T×M)

We confirmed that this provided identical results to a binomial enrichment model for integer counts of amino acids but allowed for partial assignment of peptides to clusters.

#### Quantifying the influence of sequence versus data

The magnitude of the weight used to scale the sequence and data scores is arbitrary. We do know that with a weight of 0 the model only uses the phosphorylation measurements. Alternatively, with an enormously large weight the motif information is prioritized. However, we do not know to what extent each information source is prioritized in general. Therefore, to quantify the relative importance of each type of data, we calculated our clustering results at each weighting extreme, and then calculated the Frobenius norm of the resulting peptide assignments between those and the clustering of interest.

#### Generating cluster motifs and upstream kinase predictions

For each cluster we computed a position-specific-scoring matrix (PSSM). To do so, we populated a residue/position matrix with the sum of the corresponding cluster probabilities for every peptide. Once all peptides were accounted for, the resulting matrix was normalized by averaging the mean probability across amino acids and log2-transforming to generate a PSSM. In parallel, we computed a PSSM including all sequences that served as background to account for the different amino acid occurrences within the data set. Then, we subtracted each cluster PSSM with the background PSSM to generate the final enrichment scores. Positive scores represent enriched residues while negative scores represent depleted amino acids across positions. Next, we extracted several kinase specificity profiling results (PSPL) from the literature (Miller et al., 2008; Alexander et al., 2011; Begley et al., 2015; van de Kooij et al., 2019). The distance between each cluster PSSM and kinase PSPL motif was calculated using by the Frobenius norm of the difference between both matrices, considering only positive enrichment scores. Motif logo plots were generated using logomaker (Kinney, 2019).

#### Evaluate clustering by imputation of values

To evaluate the ability of our model to handle missing values, we removed random, individual TMT experiments for each peptide and used the model to impute these values. We then computed the mean squared error between the actual values and predictions made by each method. We calculated the reconstruction error across different combinations of cluster numbers and weights using the same process.

#### Associating clusters with molecular and clinical features

To find clusters that tracked with specific molecular or clinical features we implemented two different strategies: logistic regression and hypothesis testing. For binary problems such as tumor vs NAT samples or mutational status we used l1-regularized logistic regression and the Mann-Whitney U rank test. In the former, we tried to predict the feature of interest using the phosphorylation signal of the cluster centers, whereas in the latter, for each cluster we split all patients according to their specific feature and tested whether the difference in the median signal between both groups was statistically different. We performed Bonferroni correction on the p-values computed by the Mann-Whitney U rank test. GSEA analysis was performed using clusterProfiler (4.0.2) implemented in R. The enrichment method used was “enrichWP” or “enrichGO” (WikiPathway or GeneOntology gene sets) with the p-value adjustment method was set to Bonferroni (Wu et al., 2021).

### QUANTIFICATION AND STATISTICAL ANALYSIS

All the statistical and quantification descriptions of each analysis can be found in the corresponding figure legends and results sections. The statistical enrichment of phosphorylation abundance between different binary phenotypes (tumor vs NAT, mutation vs WT, or HTE vs CTE) was calculated using the Mann-Whitney U rank test, with each subjects tumor treated as an independent observation (N = 110). The test results were adjusted for multiple tests via Bonferroni’s correction method. “*” means that the p-value is lower than 0.05 but higher than 0.001 and “**” that it is lower than 0.001. The volcano plot showing up- and down-regulated proteins in EGFR mutant vs WT samples was generated after calculating their log2 fold-change and p-values according to a Mann-Whitney U rank test using Bonferroni’s correction for multiple tests. Biokit v.2.0.8 was used to generate the volcano plot using the default log fold change and p-value cutoffs set to 1.0 and 0.05, respectively.

## Supplementary Material

1

## Figures and Tables

**KEY RESOURCES TABLE:** 

REAGENT or RESOURCE	SOURCE	IDENTIFIER

Deposited data		

LUAD phosphoproteomics, proteomics, and clinical data	Gillette et al., 2020	https://cptac-data-portal.georgetown.edu/study-summary/S056
Upstream kinase PSPLs	Begley et al., 2015; Horn et al., 2014; Miller et al., 2008; Obata et al., 2000; van de Kooij et al., 2019	https://netphorest.info/download.shtml

Software and algorithms		

Python v3.9	Python Software Foundation	https://python.org/
R	The R Foundation	https://r-project.org/
NetPhorest	Horn et al., 2014	https://netphorest.info/download.shtml
Bioinfokit 0.3	NA	https://pypi.org/project/bioinfokit/0.3/
clusterProfiler 4.2	Wu et al., 2021	https://guangchuangyu.github.io/software/clusterProfiler/
DDMC	This paper	https://doi.org/10.5281/zenodo.5856274
fancyimpute v0.5.5	NA	https://github.com/iskandr/fancyimpute

## MOTIVATION

Measuring cell signaling by mass spectrometry-based phosphoproteomics provides a promising opportunity to direct cancer therapy development. Despite continued progress in profiling the phosphoproteomes of patients across different cancer types, challenges inherent to these types of data hinder the identification of clinically relevant proteomic alterations. Here, we present DDMC: a clustering and kinase prediction strategy that identifies signaling nodes by grouping phosphosites according to their phosphorylation signal and amino acid sequence. The cluster centers, by virtue of being summaries of the phosphorylation changes of those phosphosites, can be used to establish associations with signaling responses. The sequence features of clusters can be used to identify the upstream kinases regulating them. In doing so, this method reconstructs signaling networks into biologically meaningful clusters that can be associated with cell responses and upstream kinase drivers.

## Highlights

- DDMC clusters phosphoproteomic data using the peptide’s sequence and abundance
- DDMC accurately predicts upstream kinases regulating clusters
- Clusters identify signaling specific to tumors, mutations, and immune infiltration
- DDMC provides a general and flexible strategy for phosphoproteomic analysis
