# Supplementary material for: Dual data and motif clustering improves the modeling and interpretation of phosphoproteomic data
Source: Cell Rep Methods. 2022 Feb 14;2(2):100167. doi: 10.1016/j.crmeth.2022.100167 (PMC8967184; doi:10.1016/j.crmeth.2022.100167)
Supplement: Document S1. Figures S1–S7 [file mmc1.pdf]

Cell Reports Methods, Volume *2*

Supplemental information

Dual data and motif clustering improves the modeling and interpretation of phosphoproteomic data

Marc Creixell and Aaron S. Meyer

SUPPLEMENTAL INFORMATION

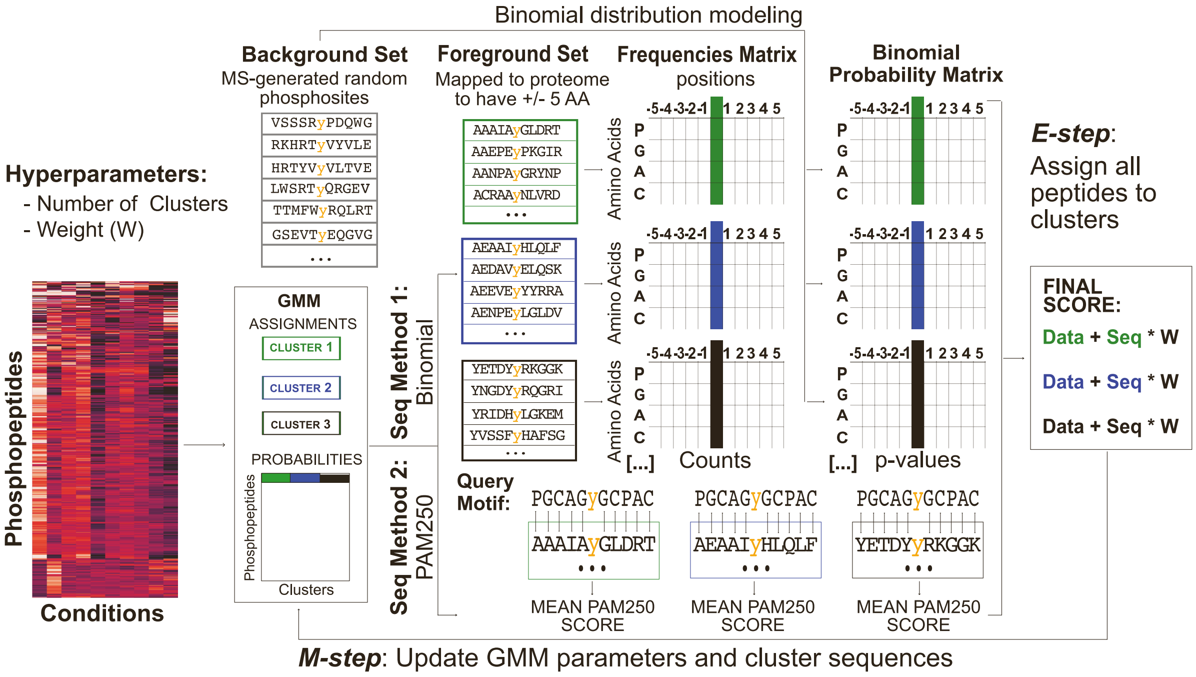

Figure S1: **Schematic of the DDMC simultaneous data and peptide sequence clustering approach.** Related to figure 1. Peptides are initialized into clusters at random. The measurements of abundance are represented by a multivariate Gaussian mixture model where each dimension of the distribution represents the abundance within a sample. Next, an expectation-maximization fitting scheme is used. During the expectation step, the distance of each peptide sequence to each cluster is calculated. This is done either through a binomial enrichment scheme (method 1, derived from (Schwartz and Gygi, 2005)) or using the average PAM250 distance (method 2). In parallel, the distance of each peptide abundance is compared to the cluster centers. These two distances are combined to update the assignments of each peptide to each cluster. During the maximization step, the cluster centers of the data are updated based on the weighted average of the peptide abundances in each condition. The peptide motifs are similarly updated through a weighted combination of the assigned peptides. Both steps continue sequentially until the change in peptide assignments between each iteration drops below a threshold.

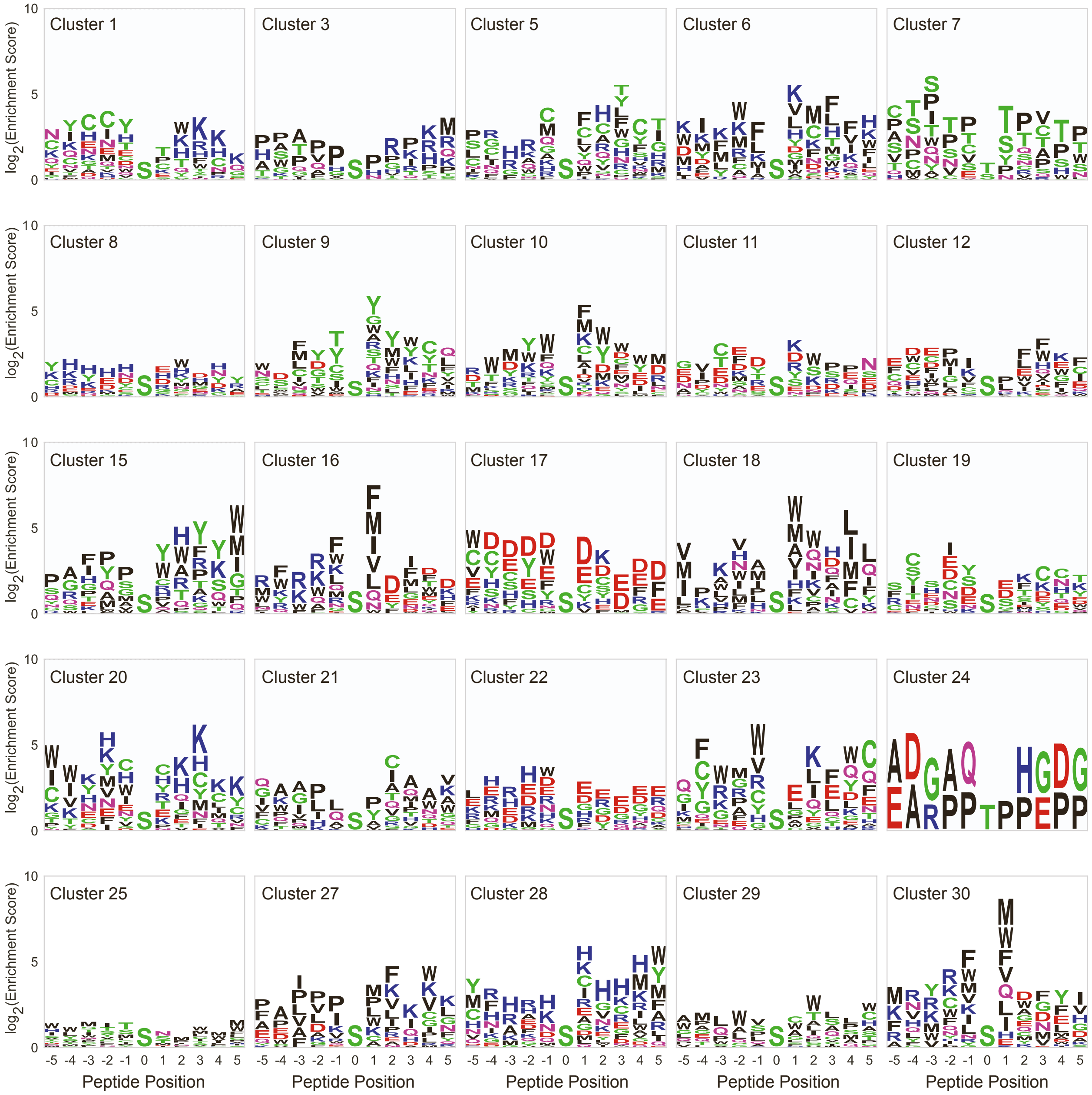

Figure S2: **Logo plots of all CPTAC Cluster PSSMs.** Related to figures 4-7. (A-U) Sequence motifs of clusters 1 through 30. Note that clusters that are not shown do not contain any peptides that are most likely assigned to them and that only 2 peptides are assigned to cluster 24. Amino acid types per color: Red=Acidic, Blue=Basic, Pink=Amidic, Black=Nonpolar, Green=polar.

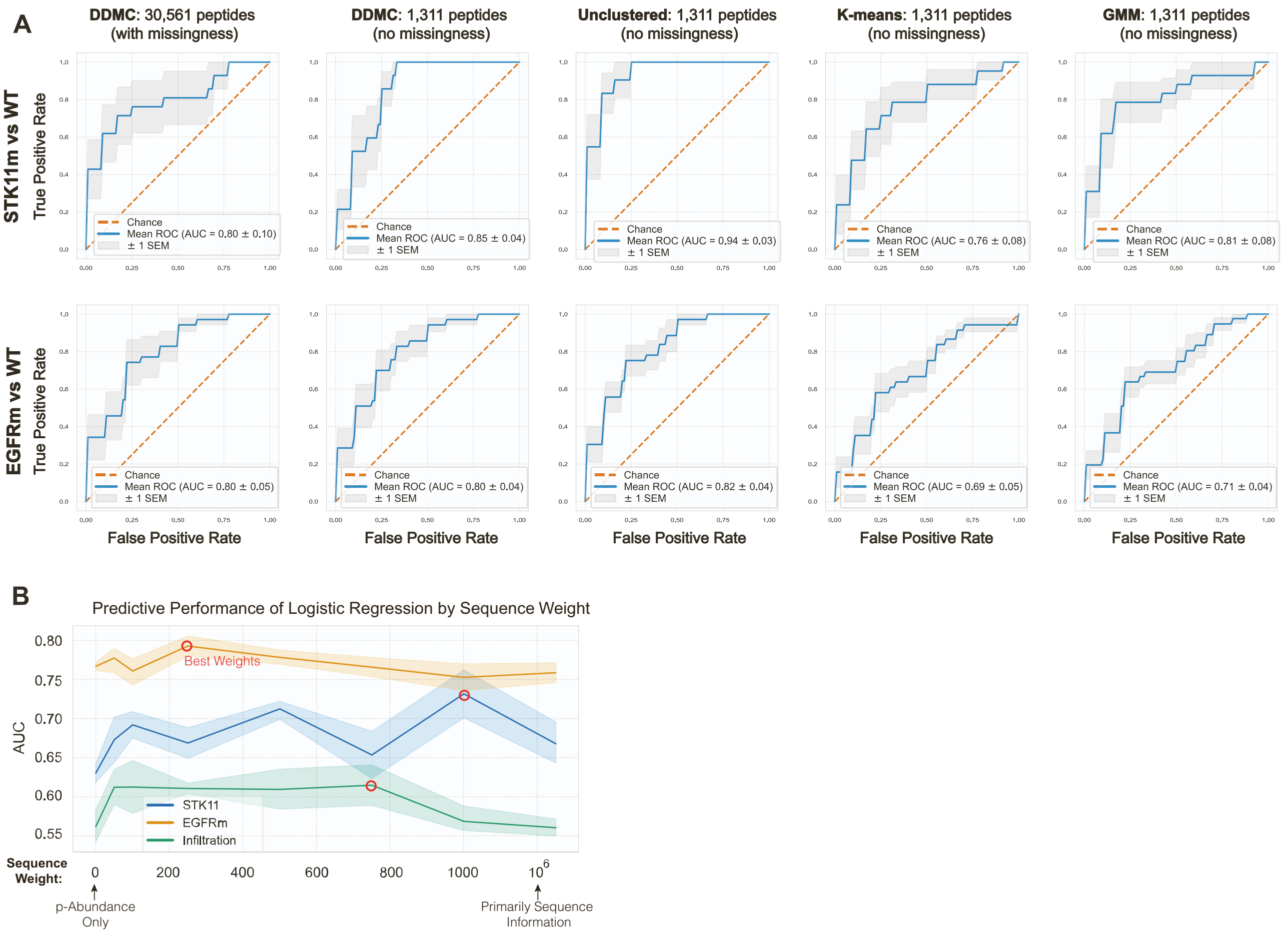

Figure S3: **Effect of sequence information on predictive performance.** Related to figure 4. A) Prediction of STK11m using either using DDMC fit to the complete data set with missingness (30,561 peptides) or the portion of the data without missingness (1,311 peptides). Using the data set without missing values, STK11m was predicted without clustering, with k-means, or GMM. The same approach was repeated to predict EGFRm. A regularized logistic regression model was used to predict the mutational statuses. B) Performance of a regression model predicting the mutational status of STK11 (blue) EGFR (yellow) and tumor infiltration level (hot versus cold) (green) in LUAD patients using either only phosphorylation data (1000,000), mainly peptide sequences (10^6), or a mix (50, 100, 250, 500, 750, 1000). Red circles denote the best predictive performance among weights.

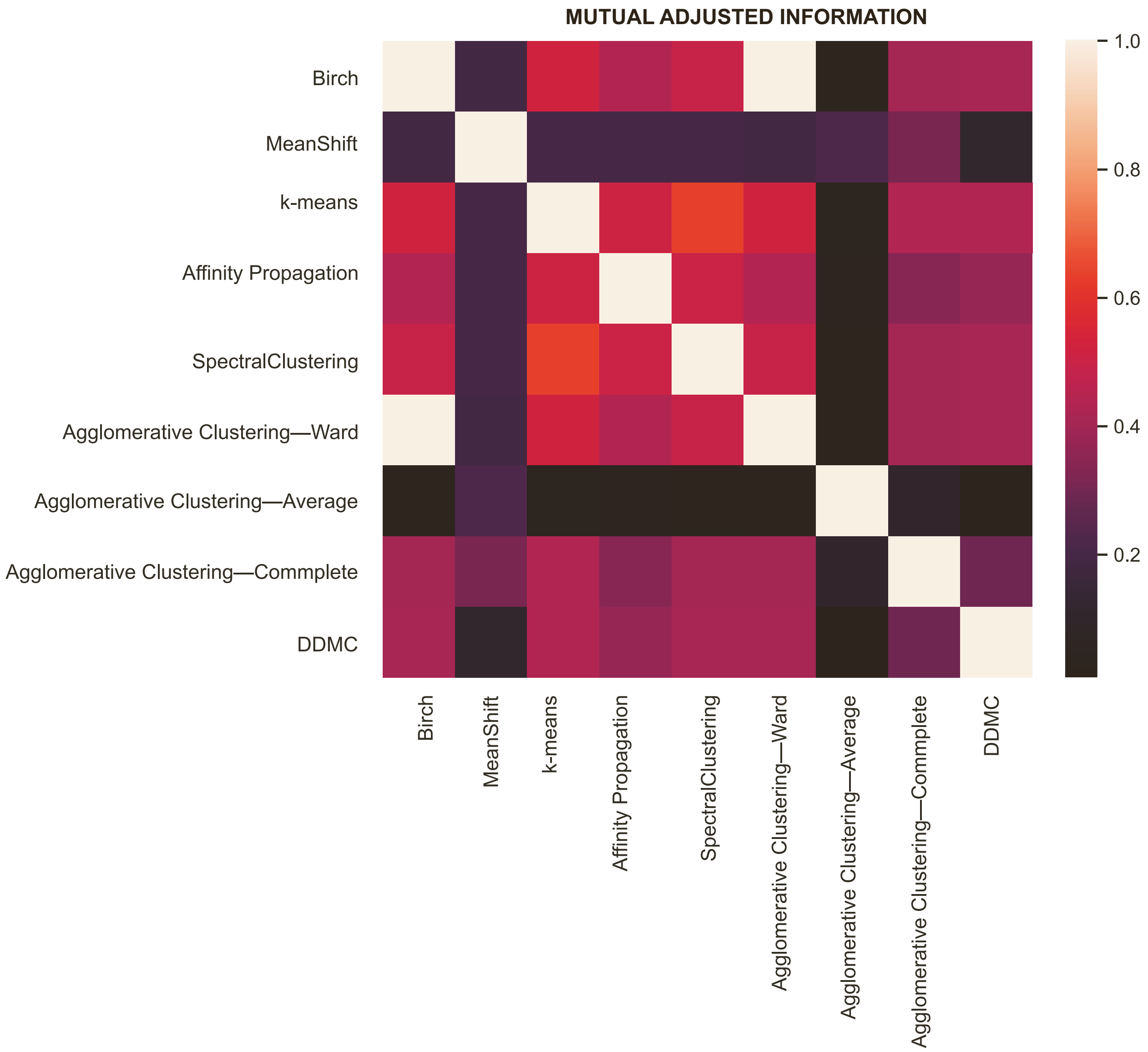

Figure S4: **Mutual adjustment information across clustering methods.** Related to figure 4. 8 different standard clustering methods and DDMC were fit to the phosphoproteomic data set generating 30 clusters. The peptide cluster assignments of each clustering method case were pairwise compared by calculating the adjusted mutual information score. Nearest neighbors was the affinity method used for spectral clustering, the remaining methods were run with the default parameters provided within scikit-learn (Zhang, Ramakrishnan and Livny, 1996; Knyazev, 2001; Comaniciu and Meer, 2002; Frey and Dueck, 2007) .

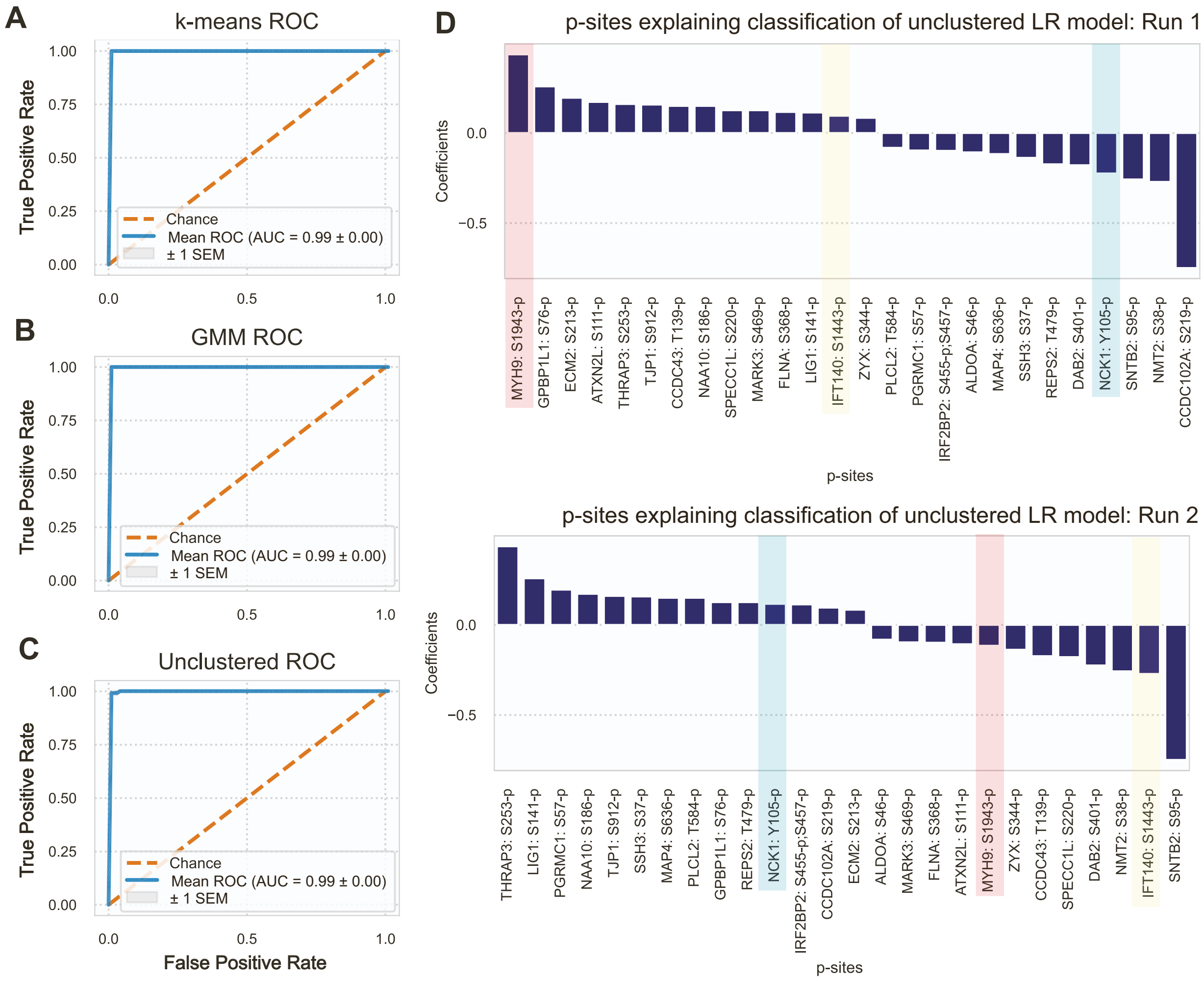

Figure S5: **Additional modeling strategies to find conserved tumor differences compared to NATs**. Related to figure 5. A-B) ROC plot of a logistic regression model fit to the complete portion of the signaling data set clustered by k-means and GMM. C-D) ROC plot of a logistic regression model fit to the complete portion of the phosphoproteomic CPTAC LUAD data set and (D) phospho-peptides with largest weights (w < 0.5 | w < -0.5) explaining the observed differences between tumors and NATs in two separate runs.

A

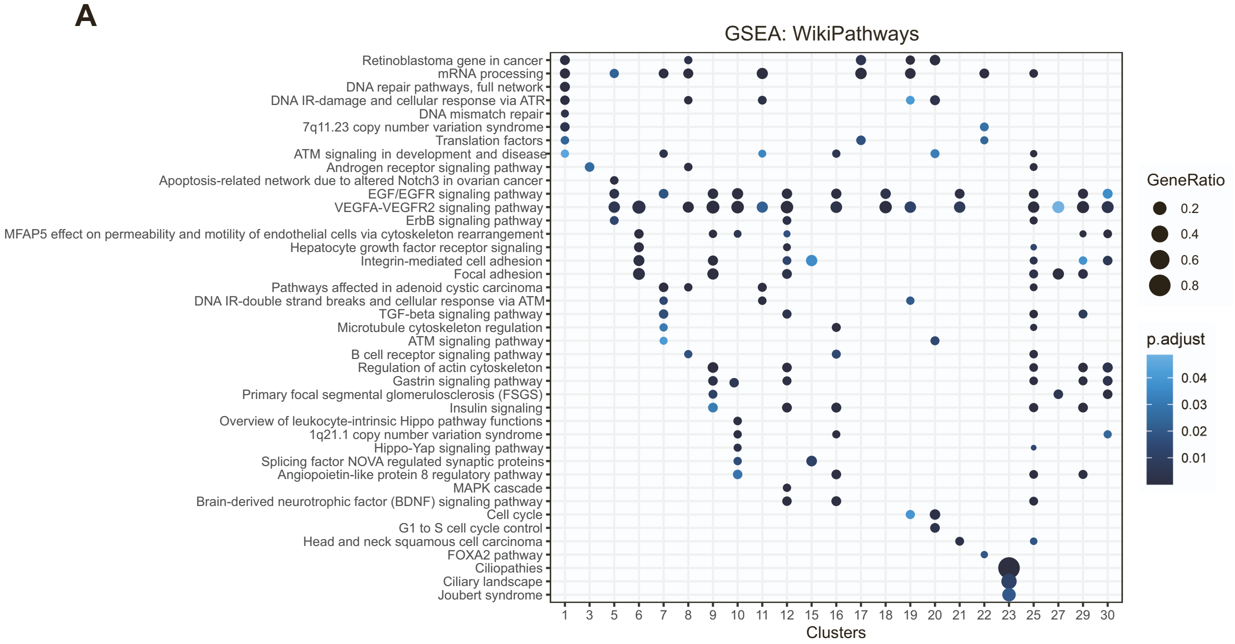

B

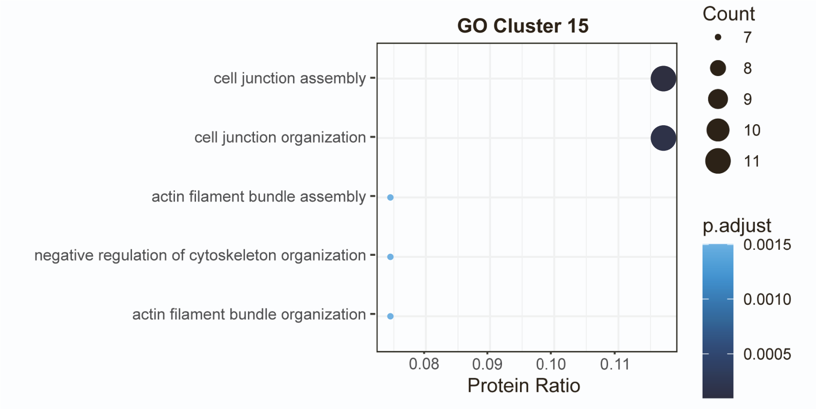

Figure S6**: Biological processes enriched in phosphoproteomic clusters**. Related to figures 5-7. A) GSEA per cluster using WikiPathway's gene set. B) Gene ontology analysis of cluster 15. GSEA was implemented using clusterProfiler (Wu et al., 2021).

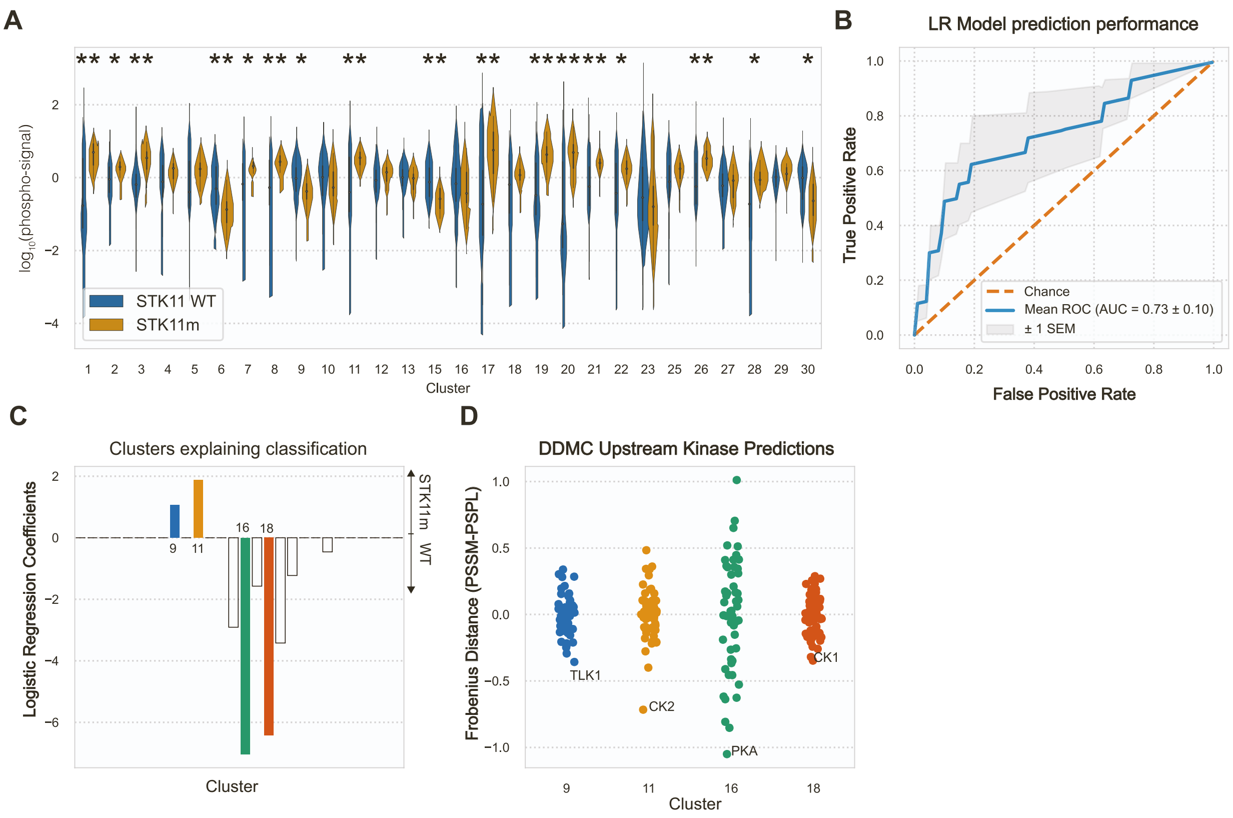

Figure S7: **Prediction of patient samples harboring STK11 mutations (STK11m)**. Related to figure 6. A) Phosphorylation signal of DDMC clusters grouped by STK11m and WT samples. Its statistical significance is indicated on the top part of the plot via a series of Mann Whitney rank test. B) ROC plot of the logistic regression model fit to the DDMC clusters. C) Logistic regression weights explaining classification of mutational status. D) Upstream kinase predictions of clusters 9, 11, 16, and 18.
